# γ-Aminobutyrate Improves the Postharvest Marketability of Horticultural Commodities: Advances and Prospects

**DOI:** 10.3389/fpls.2022.884572

**Published:** 2022-05-25

**Authors:** Morteza Soleimani Aghdam, Edward J. Flaherty, Barry J. Shelp

**Affiliations:** ^1^Department of Horticultural Science, Imam Khomeini International University, Qazvin, Iran; ^2^Department of Plant Agriculture, University of Guelph, Guelph, ON, Canada

**Keywords:** γ-aminobutyrate, biostimulants, horticultural commodities, marketability, postharvest stress

## Abstract

Postharvest deterioration can result in qualitative and quantitative changes in the marketability of horticultural commodities, as well as considerable economic loss to the industry. Low temperature and controlled atmosphere conditions (low O_2_ and elevated CO_2_) are extensively employed to prolong the postharvest life of these commodities. Nevertheless, they may suffer from chilling injury and other physiological disorders, as well as excessive water loss and bacterial/fungal decay. Research on the postharvest physiological, biochemical, and molecular responses of horticultural commodities indicates that low temperature/controlled atmosphere storage is associated with the promotion of γ-aminobutyrate (GABA) pathway activity, with or without the accumulation of GABA, delaying senescence, preserving quality and ameliorating chilling injury. Regardless of whether apple fruits are stored under low temperature/controlled atmosphere conditions or room temperature, elevated endogenous GABA or exogenous GABA maintains their quality by stimulating the activity of the GABA shunt (glutamate GABA succinic semialdehyde succinate) and the synthesis of malate, and delaying fruit ripening. This outcome is associated with changes in the genetic and biochemical regulation of key GABA pathway reactions. Flux estimates suggest that the GABA pool is derived primarily from glutamate, rather than polyamines, and that succinic semialdehyde is converted mainly to succinate, rather than γ-hydroxybutyrate. Exogenous GABA is a promising strategy for promoting the level of endogenous GABA and the activity of the GABA shunt in both intact and fresh-cut commodities, which increases carbon flux through respiratory pathways, restores or partially restores redox and energy levels, and improves postharvest marketability. The precise mechanisms whereby GABA interacts with other signaling molecules such as Ca^2+^, H_2_O_2_, polyamines, salicylic acid, nitric oxide and melatonin, or with phytohormones such as ethylene, abscisic acid and auxin remain unknown. The occurrence of the aluminum-activated malate transporter and the glutamate/aspartate/GABA exchanger in the tonoplast, respectively, offers prospects for reducing transpirational water in cut flowers and immature green fruit, and for altering the development, flavor and biotic resistance of apple fruits.

## Introduction

Fruits, vegetables and nuts are a crucial part of a healthy diet, which can help reduce risk factors for non-communicable diseases. Increasingly, consumers are concerned with the nutritional quality of these commodities (Kyriacou and Rouphael, 2018; Ziv and Fallik, 2021). Postharvest deterioration can result in qualitative and quantitative changes in their marketability, as well as incredible economic losses to the horticultural industry. Low temperature (LT) and controlled atmosphere (CA) conditions (low O_2_ and elevated CO_2_) are extensively employed to prolong the postharvest life of horticultural crops. However, horticultural crops may suffer from chilling injury and other physiological disorders, as well as excessive water loss and fungal decay (e.g., Lum et al., 2016b; Tarkowski et al., 2020; Ziv and Fallik, 2021).

The exogenous application of biostimulants, including naturally occurring plant metabolites and hormones such as polyamines (PA), salicylate, jasmonate, melatonin and γ-aminobutyrate (GABA), is being studied to improve plant tolerance/resistance to abiotic and biotic stresses under both open and closed environmental conditions (Bor and Turkan, 2019; Podlešákova et al., 2019; Akula and Mukherjee, 2020; Godoy et al., 2021; Shelp et al., 2021). The metabolism, transport, and signaling role(s) of GABA in plants were recently reviewed (Shelp et al., 2021; Xu et al., 2021b; Suhel et al., 2022). Stress-induced promotion of GABA pathways in vegetative plants, and the physiological, biochemical and molecular responses associated with enhancing stress tolerance *via* genetic manipulation of GABA metabolism and GABA receptors or the use of exogenous GABA were described (Shelp et al., 2021). Of particular interest is the demonstration that drought-induced GABA accumulation in the guard cell functions as an abscisic acid-independent mechanism for reducing stomatal reopening and transpirational water loss, thereby improving drought tolerance (Bown and Shelp, 2016; Mekonnen et al., 2016; Shelp et al., 2021; Xu et al., 2021b). GABA binds to aluminum-activated malate transporters (ALMT9/12 signaling pathway) and negatively regulates malate and/or Cl^–^ transport (Xu et al., 2021b).

This review focuses on postharvest horticultural commodities, with emphasis on botanical fruits, though some discussion of root, leaf and ornamental crops, as well as walnuts and mushrooms, is also included. First, we describe how LT and CA storage conditions improve marketability and promote GABA metabolism. Second, we discuss the genetic and biochemical control of GABA metabolism and signaling in apple fruits, and the use of exogenous GABA to preserve the postharvest quality of stored and fresh-cut horticultural commodities (i.e., delaying senescence, and enhancing resistance to chilling, browning, disease and physiological disorders) by promoting GABA shunt activity, energy generation, and antioxidant and secondary pathways. Third, we discuss prospects for enhancing postharvest drought tolerance, pathogen resistance, and flavor using exogenous GABA. Finally, we briefly comment on the safety and commercial production of GABA.

## Postharvest Marketability of Horticultural Commodities Is Linked to GABA Metabolism, and Antioxidant and Secondary Pathways

### Low Temperature and Controlled Atmosphere Storage

**FIGURE 1:**
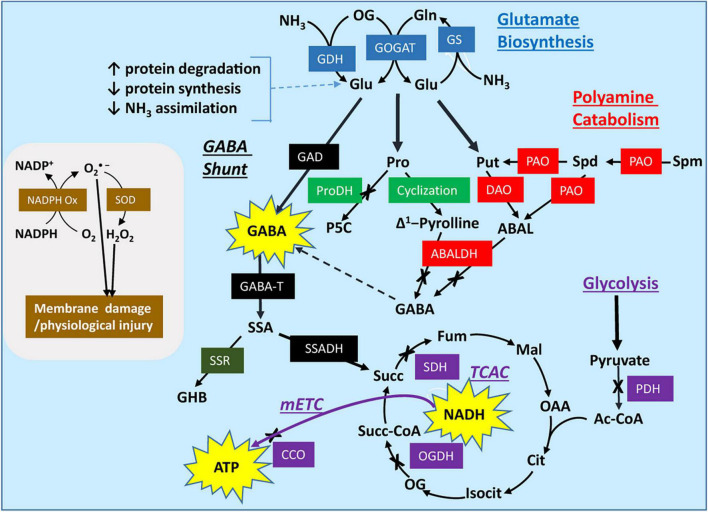
Modeling the postharvest impact of low temperature and controlled atmosphere conditions on activities of the GABA shunt, polyamine catabolism, proline catabolism, respiratory processes, and oxidant systems in horticultural commodities. Low temperature, low O_2_ and elevated CO_2_ can limit the activities of pyruvate dehydrogenase, 2-oxoglutarate dehydrogenase, succinate dehydrogenase, and cytochrome c oxidase, leading to less NADH, FADH and ATP generation and more protein turnover. This is accompanied by a shift in redox balance. The elevated NADPH/NADP^+^ ratio stimulates H_2_O_2_ production *via* NADPH oxidase and superoxide dismutase, and stimulates the expression/activities of non-enzymatic and enzymatic antioxidants (not shown). Under these conditions, the availability of Glu and the synthesis of polyamines, proline and GABA increase. Polyamines often accumulate, but evidence suggests that only about 3% of the stress-induced GABA is derived from putrescine or spermidine catabolism, which may be explained, at least in part, by O_2_ and NAD^+^ limitation of DAO, PAO, and ABALDH activities. Proline also accumulates, in part due to the decline in proline dehydrogenase activity, but there is no direct evidence for the conversion of proline into GABA *via* ABALDH (because 4-aminobutanal and △^1^-pyrroline are in rapid non-enzymatic equilibrium, their oxidation is often considered to be catalyzed by ABALDH). The limiting activities are to some extent overcome by H^+^ stimulation or Ca^2+^/calmodulin activation of glutamate decarboxylase, which increases the biosynthesis of GABA and the carbon flux through succinic semialdehyde to succinate *via* GABA transaminase and succinic semialdehyde dehydrogenase, respectively. Only a minor portion of the NADPH is recycled *via* the diversion of succinic semialdehyde into γ-hydroxybutyrate. Consequently, stress-derived succinate stimulates the production of NADH and ATP *via* the non-cyclic tricarboxylic acid cycle and the mitochondrial electron transport chain. A representative oxidant system is shown on the left; it involves NADPH oxidase and superoxide dismutase, and contributes to membrane damage and physiological injury [Please refer to Shelp et al. (2021) for more detailed graphical representations]. Symbols: ↑, increase; ↓, decrease; colored rectangles, enzymes; X, biochemical reaction potentially inhibited by stress; thick arrows, multiple biochemical steps; moderately thick arrows, the GABA shunt. ABAL, 4-aminobutanal; ABALDH, 4-aminobutanal dehydrogenase; Ac-CoA, acetyl-CoA; Cit, citrate; CCO, cytochrome oxidase; DAO, diamine oxidase; Fum, fumarate; GABA; γ-aminobutyrate; GABA-T, pyruvate/glyoxylate-dependent GABA transaminase; GAD, glutamate decarboxylase; GDH, glutamate dehydrogenase; GHB, γ-hydroxybutyrate; Glu, glutamate; GOGAT, glutamate synthase; GS, glutamine synthetase; Isocit, isocitrate; MAL, malate; mETC, mitochondrial electron transport chain; NADPH Ox, NADPH oxidase; OG, 2-oxoglutarate; OGDH, 2-oxoglutarate dehydrogenase; PAO, polyamine oxidase; Pro, proline; PDH, pyruvate dehydrogenase; ProDH, proline dehydrogenase; Put, putrescine; SDH, succinate dehydrogenase; SOD, superoxide dismutase; Spd, spermidine; Spm, spermine; SSADH, succinic semialdehyde dehydrogenase; SSR, succinic semialdehyde reductase; Succ, succinate; Succ-CoA; succinyl-CoA; TCAC, tricarboxylic acid cycle; See Table 4 legend for the remaining abbreviations.

During the postharvest storage of horticultural commodities, temperature and/or atmospheric conditions are adjusted so that ethylene production and respiratory rates are reduced, and ripening/senescence is delayed, resulting in the preservation of nutritional and sensory quality (Table 1). LT storage of mulberry leaves in air preserves color, while enhancing GABA accumulation (Li E. et al., 2018), as is often found in the vegetative organs of many plant species (Shelp et al., 2021). This result might be attributed to the elevated activity of glutamate (Glu) decarboxylase (GAD) and limited activity of the catabolic enzyme GABA transaminase (GABA-T) (Figure 1). In contrast, the LT storage of zucchini fruit promotes GABA catabolism, without causing its accumulation (Palma et al., 2014). The loss of GABA and the increase in GABA-T activity is more substantive in a chilling-tolerant cultivar than a chilling-sensitive cultivar, suggesting that GABA catabolism replenishes the tricarboxylic acid cycle (TCAC) to generate reducing equivalents and energy that could alleviate oxidative damage (Shelp et al., 2021). The authors have interpreted the increase in diamine oxidase (DAO) activity and putrescine (Put) accumulation as support for the involvement of Put catabolism in GABA production and the alleviation of chilling injury (Figure 1). Conditioning at 15°C prior to LT storage improves the tolerance in the chilling-sensitive zucchini cultivar by decreasing the GABA level and increasing the ATP level and activities of enzymatic antioxidants (peroxidase, catalase) (Carvajal et al., 2015). Improved chilling tolerance in peaches by hot water treatment prior to LT storage is associated with membrane stability (as indicated by less electrolyte leakage and lower malondialdehyde accumulation), and the maintenance of high levels of amino acid (including GABA and proline), polyamines (PAs) and radical scavenging capacity (phenols) (Wang L. et al., 2021).

**TABLE 1:** Postharvest storage conditions improve the marketability of horticultural commodities and promote GABA metabolism.

Commodity	Storage conditions	Marketability	Biochemical and molecular responses	References
Mulberry leaves (*Morus alba* L.)	4°C, air for 5 days	Preserves color	•↑ GABA, GAD activity; ↓ GABA-TP activity	Li E. et al., 2018
Zucchini fruit (*Cucurbita pepo* L.)	4°C, air for 14 days	Preserves FM	•↓ GABA; ↑ Pro, free Put, and conj soluble Put •↑ activities of GABA-T and DAO	Palma et al., 2014
Peach fruit (*Prunus persica* L.)	Hot water at 45°C for 10 min, then stored at 0°C for 35 days	Attenuates chilling injury	•↑ GABA, arginine, Pro, Put, Spd, and Spm •↑ Expression of *GAD1,4*, *GABA-T3, ARG*, *P5CS, OAT, ADC, ODC, PAL1*, and *4CL*, and corresponding activities •↓ Expression of *ProDH, DAO* and *PAO1,4,5*, and corresponding activities •↓ EL and MDA	Wang L. et al., 2021
Green tea leaves (*Commelia sinesis* [L.] O. Kuntze)	RT, 100 kPa N_2_ during drying	Preserves quality	•↑ GABA, alanine, and GHB	Allan et al., 2003
Soybean sprouts (*Glycine max* [L.] Merr.)	RT, 100 kPa N_2_ for 100 h	Preserves quality	•↑ GHB	Allan et al., 2003
Green tea leaves (*Commelia sinesis* [L.] O. Kuntze)	25°C, 100 kPa N_2_ for 6 h	Induces GABA accumulation	•↑ GABA; ↑ expression of *GAD2*, *GLYR1*, and *GDH1*; ↓ Glu • Expression of *GAD1,3, GABA-T1,2, SSADH1,2*, *GLYR2*, and *GDH2* unaffected • N_2_ activates CaM-dependent GAD1 • N_2_ and mechanical stress (i.e., picking) induce CaM-independent *GAD2*	Mei et al., 2016
	25°C, 100 kPa N_2_ for 11 h	Induces GABA accumulation	•↑ GABA, Put, Spm, and Spd; transient ↑ GAD activity; ↑ DAO activity; ↑ expression of *GAD1*,*2,3* •↓ Glu	Liao et al., 2017
	25°C, 100 kPa N_2_ or CO_2_ for 6 h	Preserves quality	•↑ GABA, Succ, Pro, and Put greater with CO_2_ than N_2_ •↑ Glu, alanine, and pyruvate, and ↓ citrate, 2-OG and fumarate more with N_2_ than CO_2_	Chen et al., 2018
Mushroom [*Agaricus bisporus* (J.E. Lange) Imbach]	4°C, 100 kPa N_2_ or CO_2_ for 1 day	Preserves quality	•↑ GABA; ↑ activities of GAD, GABA-TP, and PAO; ↓ Put, Spd, and Cad; may ↑ activities of ADC, ODC, PAO, and DAO •↑ GABA and alanine, ↓ Arg, Orn, and DAO activity more with N_2_ than CO_2_ •↑ Glu with CO_2_; ↓ Glu with N_2_	Chen et al., 2020
Broccoli florets (*Brassica oleracea* var. *italica* Plenck)	10°C, 20 kPa CO_2_ + N_2_ for 7 days	Delays senescence	•↑ GABA and non-protein AAs •↓ Glu, aspartate and protein AAs •↓ GABA with re-aeration for 2 days	Hansen et al., 2001
Red tomato fruit (*Solanum lycopersicum* L.)	30°C, 11 kPa O_2_ + 11 kPa CO_2_ for 6 days	Delays ripening	↑ GABA, and GAD activity ↓ GABA-TOG activity; GABA-TP activity unaffected	Makino et al., 2008
	25°C, 2.4–3.5 kPa O_2_ + 10 kPa CO_2_ for 7 days	Delays ripening	•↑ GABA; alanine and Glu unaffected •↑ GAD activity, expression of *GAD1,2,3* •↓ GABA-TOG activity.	Mae et al., 2012
	13°C, 10 kPa CO_2_ in air for 12 days	Delays ripening	•↑ GABA, and *GAD2,3* expression •↓ Activities of GABA-TP, and SSADH; ↓ expression of *GABA-T1*, and *GYR1,2* • GAD activity and expression of *GAD1*, *GABA-T2,3*, and *SSADH* unaffected •↓ GABA, ↑ *GABA-T2,3* expression upon re-aeration for 3 days	Deewatthanawong et al., 2010b
	20°C, 20 kPa CO_2_ in air for 3 days	Delays ripening	•↑ Expression of *GAD*, and *HSP* •↓ Ethylene; ↓ expression of *ACS*, *ACO*, *PSY, PG*, and *INV* •↓ *GAD* expression, ↑ expression of *ACS* and *ACO* upon re-aeration for 4 days	Rothan et al., 1997
Cherimoya fruit (*Annona cherimola* Mill.)	6°C, 20 kPa CO_2_ in air for 3 days	Improves chilling tolerance and preserves quality	•↑ GABA, and total PA •↓ GABA, and total PA upon re-aeration for 3 days	Merodio et al., 1998
Strawberry fruit (*Fragaria* × *ananassa* Duch.)	2°C, 20 kPa CO_2_ in air for 12 days	Delays ripening and preserves fruit color.	•↑ GABA • GAD activity unaffected, but GABA-TP activity may ↓, depending upon cv	Deewatthanawong et al., 2010a
	0°C, 20 kPa CO_2_ in air for 12 days	Preserves quality and alleviates decay	•↑ GABA, NAD^+^; ↑ SDH and CCO activities •↓ *GABA-TP* expression and activity, AEC, ATP, NADH, and NADH/NAD^+^ • GAD activity, expression of *GAD1* and *GABA-T1* unaffected	Li D. et al., 2018
	4°C, 10 kPa CO_2_ + 11 kPa O_2_ for 10 days	Preserves quality	•↓ GABA, Pro, OG •↑ Fumarate, and Succ	Pott et al., 2020
Strawberry fruit (*Fragaria vesca* L.)	0°C, 20 kPa CO_2_ in air for 3 days	Preserves FM and cell structure	•↑ GABA (CO_2_-independent), Glu, alanine, Pro, Succ, oxalate, and sugars •↓ Malate; citrate unaffected	Blanch et al., 2012
Longan fruit (*Dimocarpus longan* Lour.)	4°C, 5 kPa O_2_ + 5 kPa CO_2_ for 18 days	Delays senescence	•↓ GABA, and GAD activity • GABA-T activity fluctuates	Zhou et al., 2016
Peel from apple fruit (*Malus* × *domestica* Borkh. “Empire”)	3°C, 2 kPa O_2_ + 5 kPa CO_2_ for 4 weeks	Delays senescence, but induces external injury	•↑ GABA	Deewatthanawong and Watkins, 2010
Apple fruit (“Empire”)	3°C, 2.5 kPa O_2_ + 2.5 kPa CO_2_	Delays senescence	•↓ GABA upon aeration for 3 h	Trobacher et al., 2013a,b
	3°C, 2.5 kPa O_2_ + 2.5 kPa CO_2_ for 46 weeks	Delays senescence	•↑ GABA; ↓ Glu after a transient peak •↑ Total PAs, including free and soluble/insoluble conjug forms	Deyman et al., 2014a
	3°C, 2.5 kPa O_2_ + 5 kPa CO_2_ for 16 weeks	Delays senescence, but increases external injury	•↑ GABA, alanine, Succ, GHB, Put, Spd, and Spm; ↓ Glu (short-term) •↑ Expression of *GAD2*, *AO1*, *ALDH10A8* and *PAO2* (long-term, CO_2_-dependent) •↓ NADH (short-term) and NADH/NAD^+^ (long-term); ↑ NADPH (long-term) and NADPH/NADP^+^	Deyman et al., 2014b; Brikis et al., 2018
Apple fruit (“Honeycrisp”)	3°C, 2.5 kPa O_2_ + 5 kPa CO_2_ for 24 weeks	Increases CA-related injury by 24 weeks	•↑ GABA from 18 to 24 weeks	Chiu et al., 2015
	Conditioned at 10°C in air for 5 days, followed by 3°C in 2.5 kPa O_2_ + 2.5 CO_2_ for 35 weeks	Delays onset of CA-related injury	•↓ GABA	Lum et al., 2016a
Pear fruit (*Pyrus communis* L.)	0°C, air for 167–180 days	Delays senescence	•↑ GABA from 111–119 days to 167–180 days, depending on cv	Lum et al., 2017

*Symbols: ↑, increases; ↓, decreases.*

*ACO, 1-aminocyclopropane-1-carboxylic acid oxidase; ACS, 1-aminocyclopropane-1-carboxylic acid synthase; ADC, arginine decarboxylase; AEC, adenylate energy charge; AA, amino acid; AO, Cu amine oxidase; ARG, arginase; 4CL, 4-coumarate/coenzyme A ligase; CA, controlled atmosphere; Cad, cadaverine; CaM, calmodulin; CCO, cytochrome c oxidase; conj, conjugated; cv, cultivar; DAO, diamine oxidase; EL, electrolyte leakage; GABA, γ-aminobutyrate; GABA-TP or GABA-TOG, pyruvate/glyoxylate or 2-oxoglutarate-dependent GABA transaminase; GAD, glutamate decarboxylase; GHB, γ-hydroxybutyrate; Glu, glutamate; GLYR, glyoxylate/succinic semialdehyde reductase; INV, acid invertase; MDA, malondialdehyde; NAD^+^/NADH, oxidized/reduced dinucleotide; NADPH, reduced dinucleotide phosphate; OAT, ornithine δ-aminotransferase; ODC, ornithine decarboxylase; 2-OG, 2-oxoglutarate; P5CS, Δ^1^-pyrroline-5-carboxylate synthetase; PA, polyamine; PAL, phenylalanine lyase; PAO, polyamine oxidase; PDC, pyruvate decarboxylase; Pro, proline; ProDH, proline dehydrogenase; PG, polygalacturonase; Pro, proline; PSY, phytoene synthase; Put, putrescine; RT, room temperature; SDH, succinate dehydrogenase; Spd, spermidine; Spm, spermine; SSADH, succinic semialdehyde dehydrogenase; Succ, succinate; TCAC, tricarboxylic acid cycle; TAA, total amino acids.*

Anoxia preserves the quality of drying green tea leaves and of soybean sprouts stored at room temperature (RT) and promotes GABA accumulation and the diversion of succinic semialdehyde (SSA) from succinate to γ-hydroxybutyrate (GHB) (Allan et al., 2003; Table 1 and Figure 1). Several mechanisms could account for the accumulation of GABA: calmodulin (CaM) activation of CsGAD1; elevated expression of *CsGAD2*; oxidation of Put/proline; and, feedback inhibition of GABA transaminase (*Cs*GABA-T) activity (Mei et al., 2016; Liao et al., 2017; Shelp et al., 2021; Table 1). Complete inhibition of DAO activity by aminoguanidine (4–11 h of treatment) suggests that about 25% of the GABA is derived from the PA degradation pathways (Liao et al., 2017; Figure 1). However, this interpretation can be challenged. Based upon the increasing accumulation of Put with aminoguanidine over the same time period, we estimate that Put degradation would account for only 3% of the anoxia-induced rate of GABA accumulation. Notably, the spermidine (Spd) pool also decreases at an estimated rate of approximately 3% of the rate for GABA accumulation, suggesting that the terminal oxidation of Spd can substitute for the terminal oxidation of Put (Shelp et al., 2012b). This re-assessment of the published data is consistent with our recent interpretation of the ΔGABA/ΔPut stoichiometry published for wheat roots treated simultaneously with salinity and aminoguanidine (Shelp et al., 2021). While increasing DAO activity seems contrary to our interpretation of the metabolite data, it could reflect an “anticipation response” to the return to normoxia, as proposed for alanine transaminase and glutamate dehydrogenase (Limami et al., 2008).

The quality of green tea leaves at RT and of broccoli florets at LT is preserved under anaerobic conditions imposed by either anoxia or elevated CO_2_ (Hansen et al., 2001; Chen et al., 2018; Table 1). However, the accumulation of GABA and succinate, and the depletion of Glu is more rapid with CO_2_ than with N_2_, whereas the accumulation of alanine is faster with N_2_. There is greater Put and NADH accumulation, and less NADPH, citrate, 2-oxoglutarate (OG) and fumarate accumulation with CO_2_ than air. Thus, the GABA shunt is more active with CO_2_, but the inhibition of the TCAC and mitochondrial electron transport chain (mETC) occurs more quickly with N_2_ (Chen et al., 2018). While the storage of mushrooms with 100% CO_2_ at LT also stimulates the production of Glu-derived GABA, storage with N_2_ stimulates the production of both Glu-and PA-derived GABA (Chen et al., 2020), perhaps due in part to protein degradation. Notably, *Ab*GAD, unlike most plant GADs, does not possess a CaM-binding domain, and therefore its activity is likely to be stimulated by cytosolic acidification only.

Elevated CO_2_ in air at LT improves chilling tolerance in cherimoya fruit (Merodio et al., 1998), and delays ripening/senescence in tomato and strawberry fruits (Deewatthanawong et al., 2010a,b; Blanch et al., 2012; Li D. et al., 2018; Table 1). These positive outcomes are typically accompanied by the accumulation of GABA and occasionally PAs, as well as limited flux of GABA-carbon through the GABA shunt into the TCAC and the mETC. Elevated CO_2_, in combination with low O_2_, delays the ripening/senescence of tomato fruit stored at RT (Makino et al., 2008; Mae et al., 2012), and longan (Zhou et al., 2016), strawberry (Pott et al., 2020) and “Empire” apple (Deewatthanawong and Watkins, 2010; Trobacher et al., 2013a; Deyman et al., 2014a,b; Brikis et al., 2018) fruits stored at LT. These findings have been attributed to the elevated generation of GABA from Glu, rather than PAs, and enhanced flux of GABA-carbon through the GABA shunt into a non-cyclic TCAC for generation of ATP (Shelp et al., 2012b; Brikis et al., 2018). Wang C. et al. (2014) previously suggested that Glu-derived GABA accumulation in melon roots can alleviate hypoxia damage by accelerating PA biosynthesis and conversion, as well as preventing PA degradation.

Some pome fruit are particularly sensitive to LT, CA storage (e.g., “Honeycrisp” apples and “Cold Snap” pears) (Chiu et al., 2015; Lum et al., 2016a,^2017^; Table 1). In these cases, a dramatic increase in the GABA level coincides with CA-or senescence-related injury and is likely due to the disruption of cellular compartmentation and the release of acidic vacuolar contents to the cytosol (Bown and Shelp, 2006). Interestingly, conditioning of “Honeycrisp” apples at 10°C improves the resistance to CA-related injury, decreases the GABA level and increases the ratios of NAD(P)H/NAD(P) ^+^ (Lum et al., 2016a).

Overall, these studies indicate that LT, CA-mediated improvements in the postharvest marketability of horticultural products is generally associated with the promotion of GABA biosynthesis and GABA shunt activity, with or without the accumulation of GABA. The onset of CA-or senescence-related injury during prolonged storage may also be associated with the accumulation of GABA. Discrepancies in data from the various studies might be explained by: pretreatment and conditioning of plant materials prior to storage; the use of different cultivars and single time point determinations, rather than time courses; excessive handling or wounding of plant materials prior to metabolite extraction; and, the use of non-saturating levels of Glu and inhibitory levels of GABA and pyruvate, respectively, in *in vitro* assays of GAD and GABA-TP activities [for examples, compare Zhou et al. (2016), Li D. et al. (2018), and Chen et al. (2020) with Snedden et al. (1995), Van Cauwenberghe and Shelp (1999), Clark et al. (2009a), and Shelp et al. (2012a)]. Interpretation could vary somewhat because: (i) it is not possible to directly assess the Ca^2+^/CaM stimulation of GAD activity *in situ*; (ii) the existence of a 2-OG-dependent plant GABA-T is questionable [for examples, compare Makino et al. (2008) and Deewatthanawong et al. (2010b) with Clark et al. (2009b), Koike et al. (2013), Trobacher et al. (2013a), Shimajiri et al. (2013), and Shelp et al. (2021)]; (iii) the expression of a gene does not establish that the encoded protein is operational; (iv) the understanding of precursor/product relations and flux is often incomplete (e.g., pool sizes alone do not indicate flux; Put accumulation does not establish greater contribution than Glu to GABA generation) (Shelp et al., 2012a); and, (v) there is often a failure to consider the importance of multiple isoforms of the GABA pathway enzymes (Shelp et al., 2012c,^2021^).

### Genetic and Biochemical Control of GABA Metabolism in Apple Fruits

Gene sequences for the key steps in GABA metabolism in apple fruit have been identified, allowing elucidation of the biochemical properties and subcellular location of multiple isoforms of the encoded proteins (Table 2 and Figure 1). Three cytosolic GADs are present, but unlike *Md*GAD3, *Md*GAD1,2 are Ca^2+^/CaM-dependent and more sensitive to pH (Trobacher et al., 2013b). There are also two mitochondrial pyruvate/glyoxylate-dependent GABA transaminases (*Md*GABA-Ts, designated as GABA-TP), two mitochondrial NAD^+^-dependent SSADHs (*Md*SSADH1,2 or *Md*ALDH5F1,2), and two NADPH-dependent glyoxylate/succinic semialdehyde reductases (*Md*GLYR1,2 or *Md*SSR1,2) with different subcellular locations (Trobacher et al., 2013a; Brikis et al., 2017, 2018; Zarei et al., 2017). *Md*GLYR1 is cytosolic, whereas *Md*GLYR2 is both plastidial and mitochondrial. Two of the six apple fruit FAD-dependent polyamine oxidases (*Md*PAO2,4) are peroxisomal and likely catalyze the back-conversion of Spm and Spd to Spd and Put, respectively (Brikis et al., 2018). Three of the five *Md*CuAOs identified are peroxisomal (*Md*CuAO1,4-5), but only one of these, *Md*CuAO1, has been shown to exclusively utilize diamines (diaminopropane, Put and cadaverine) as substrates (Zarei et al., 2015a; Brikis et al., 2018). A candidate plastidial diamine oxidase activity has not yet been identified. Two NAD^+^-dependent 4-aminobutanal dehydrogenases (ABALDH) exist in apple fruit (*Md*ALDH10A8,9 or *Md*AMADH1,2): one is peroxisomal and the other plastidial (Zarei et al., 2015b,^2016^; Brikis et al., 2018).

**TABLE 2:** Key proteins/genes of GABA metabolism and signaling in apple fruits subjected to low temperature, controlled atmosphere storage.

Protein/ gene name	Accession number	Subcellular location	Expression profile	References
**MdGAD1^a^**	KC812242	C^b^	U	Trobacher et al., 2013b; Brikis et al., 2018
**MdGAD2**	KC812243	C^b^	U	
MdGAD3	KC812244	C^b^	D	
**MdGABA-T1**	JX276380	M	U	Trobacher et al., 2013b; Brikis et al., 2018
**MdGABA-T2**	JX276381	M	U	
**MdSSADH1**	XM_008357890	M^b^	U	Brikis et al., 2018; Jung et al., 2019
MdSSADH2	XM_029110087 ^c^	M^b^	D	
MdGABP	XM_008341399	M^b^	–	Jung et al., 2019
**MdSSR1**	KT202799	C	TU	Brikis et al., 2017, 2018; Zarei et al., 2017
**MdSSR2**	KT202800	P/M	TU	
**MdPAO2**	KT184497	Px^b^	U	Brikis et al., 2018
**MdPAO4**	KT184499	Px^b^	U	
**MdCuAO1**	KM067895	Px	U	Zarei et al., 2015a; Brikis et al., 2018
MdCuAO4	KM067898	Px^b^	TU	
MdCuAO5	KM067899	Px^b^	TU	
**MdALDH10A8**	KP218041	P^b^	U	Zarei et al., 2015b; Zarei et al., 2016; Brikis et al., 2018
**MdALDH10A9**	KP218040	Px	U	
MdCAT9	XM_008368457	T^b^	–	Shelp and Zarei, 2017; Jung et al., 2019
MdALMT9	MDP0000252114	T^b^	–	Li et al., 2020

*ES, extracellular/secretory pathway; U, upregulated; D, downregulated; T, tonoplast; TU, transiently upregulated; C, cytosol; M, mitochondrion; P, plastid; Px, peroxisome.*

*^a^Proteins in bold lettering are likely to be the most abundant of the alternative forms (based on gene transcript abundance).*

*^b^Predicted.*

The temporal patterns of specific metabolites have been compared to the expression of genes encoding the most biochemically relevant proteins in intact “Empire” apple fruit stored under LT and low O_2_ with ambient or elevated CO_2_ (0°C, 2.5 kPa O_2_ and 0.03 or 5 kPa CO_2_) (Brikis et al., 2018; Table 2). Five kPa CO_2_ is known to elicit symptoms of external, but not internal, CO_2_-induced injury in this cultivar within 16 weeks (Deyman et al., 2014b). Under LT, low-O_2_ and ambient-CO_2_ storage, there is a transient increase in amino acid availability, including Glu, early in the storage period (2–4 weeks), probably a reflection of protein hydrolysis (Brikis et al., 2018). This is accompanied by a rapid peak in the expression of *alanine transaminase* (*MdAla-T*), a marker of hypoxia (Cukrov et al., 2016), as well as in the pool of alanine (2–4 weeks), which decline slowly to a steady basal level (from 8 to 16 weeks) (Brikis et al., 2018). A rapid accumulation of GABA is also transient (2–4 weeks), but the pool size is approximately 60% of that for alanine, suggesting that the alanine is derived from both Ala-T and GABA-TP reactions. Notably, *MdGAD1* expression increases linearly up to 12 weeks and then remains steady, whereas *MdGAD2* expression increases up to only 4 weeks and then decreases, and *MdGAD3* expression decreases over the storage period. Succinate does not accumulate, but the burst of GABA is followed by a much smaller transient increase in GHB (Figure 1). Nevertheless, *MdSSADH1* expression increases up to 8 weeks and then slowly declines, whereas *MdSSADH2* expression decreases over the storage period. The expression of *MdSSR1* is transiently increased, peaking at 4–8 weeks, and may be correlated with GHB. While Put, Spd and spermine (Spm) represent potential precursors for GABA, their levels are only 1–5% of that for GABA. Furthermore, the Put level declines only slightly with the increase in GABA, while Spd accumulates slightly and Spm dramatically declines. The expression of *MdPAO2,4, MdCuAO1* and *MdALDH10A8,9* rapidly increases, peaking after 8, 2 and 4–8 weeks, respectively, whereas the expression of *MdCuAO4-*5 is transiently increased, peaking at 4 weeks.

With LT, low-O_2_ and elevated-CO_2_ storage, a pronounced transient peak of GABA is accompanied by a strong transient peak of succinate, and smaller transient peaks of GHB, Put, Spd and Spm (Brikis et al., 2018). With prolonged storage, only GABA and GHB exhibit subsequent increases. These changes are accompanied by minor, yet significant, increases in the expression of *MdGAD1*, *MdCuAO1* and *MdALDH10A8,9*. Thus, the GABA pattern might be interpreted as a CO_2_-induced shift from Glu/CaM-mediated stimulation/activation of GAD activity to H^+^-mediated stimulation of GAD activity (Trobacher et al., 2013b; Brikis et al., 2018). The patterns for succinate, GHB and Put might be explained by a combination of: elevated GABA production; differential effects of shifting redox balance on the activities of SSADH, TCAC, SSR, and ABALDH; and, limiting O_2_ availability for DAO activity in bulky apple fruit (Shelp et al., 2012b; Brikis et al., 2018). Based on changing pool sizes, we can estimate the maximum rates of GABA and succinate synthesis to be ∼50 nmol g^–1^ fresh mass (FM) wk^–1^, and the maximum rates of GHB synthesis and Put/Spd depletion to be ∼0.2 and ∼1.5 nmol g^–1^ FM wk^–1^, respectively. Thus, the terminal oxidation of PAs and the direct decarboxylation of Glu can account for approximately 3 and 97%, respectively, of GABA synthesis. Moreover, only 3% of the SSA is diverted from succinate to GHB production. Overall, this study suggests that both genetic and biochemical mechanisms are involved in the metabolism of GABA in apple fruit stored under LT, CA conditions.

Han et al. (2018) have monitored the expression of the GABA shunt enzymes and the levels of important metabolites in “Cripps Pink” apple fruit stored at RT in air for 70 days. The *MdGAD*s exhibit different expression patterns, with *MdGAD1* expression increasing gradually with time, *MdGAD2* expression increasing until 30 days and then decreasing, and *MdGAD3* expression decreasing. The expression of *MdGABA-T1,2* and *MdSSADH1* increases gradually from 0 to 30 days, peaking at the same time as the ethylene climacteric peak (30 days). Thus, the expression of *MdGAD1*, *MdGAD2*, *MdGABA-T1,2*, and *MdSSADH1* in “Cripps Pink” apple fruit responds strongly under RT storage, essentially as in “Empire” apple fruit stored under LT, low O_2_ and ambient or elevated CO_2_ (Brikis et al., 2018). These findings, in conjunction with those of Brikis et al. (2018), lead us to conclude that the postharvest expression patterns for GABA shunt genes in apple fruits are more influenced by development, than by environment.

The temporal patterns for GABA (i.e., slow decrease of approximately 60% from 10 to 40 days, followed by a dramatic increase at 70 days, presumably due to fruit aging and cellular disintegration at the end of storage), succinate and malate (slow decrease of 40 and 20%, respectively, from 30 to 70 days) indicate that GABA does not accumulate under storage at RT, and that GABA is probably catabolized to succinate and malate (Han et al., 2018). The application of exogenous GABA increases the expression of *MdGAD1*, *MdGAD2, MdGABA-T1,2* and *MdSSADH1*, restrains the decrease in malate and succinate levels, decreases respiration and ethylene production rates, and delays the ethylene production peak (Han et al., 2018; Table 3). Notably, the application of exogenous Ca^2+^ decreases the Glu level (before 30 days), increases the levels of GABA, succinate and malate (10–60 days) and expression of *MdGAD1* (before 30 days), *MdGAD2* (20–40 days), *MdGABA-T1/2* (10–20 days) and *MdSSADH* (20–40 days), suppresses the respiration rate, and decreases the ethylene production peak (Han et al., 2021).

**TABLE 3:** The application of exogenous GABA improves the postharvest marketability of horticultural commodities by promoting GABA and antioxidant pathways.

Commodity	Storage conditions	Marketability	Biochemical and molecular responses	References
Peach fruit (*Prunus persica* L.)	1°C, 5 weeks	Chilling tolerance	•↑ GABA, Pro, ATP, and ADP; ↑ activities of GAD, P5CS, OAT, SOD, CAT, APX, GPX, GST, GR, DHAR, and MDHAR •↓ AEC, and ProDH activity	Shang et al., 2011; Yang et al., 2011
Banana fruit (*Musa* spp. Cavendish)	7°C, 20 days	Chilling tolerance	•↑ Pro, and phenols; ↑ activities of P5CS, PAL, DPPH and FRAP scavenging capacity •↓ PDH activity, MDA, and EL	Wang Y. et al., 2014
Zucchini fruit (*Cucurbita pepo* L.)	4°C, 14 days	Chilling tolerance	•↑ Pro, malate, fumarate, ATP, and NADH; ↑ GABA-TP activity	Palma et al., 2019
Orange fruit [*Citrus* × *sinensis* (L.)]	3°C, 120 days	Chilling tolerance	•↑ ASC, phenols, and anthocyanins; ↑ activities of SOD, CAT, and APX; ↑ PAL/PPO activity ratio, and DPPH scavenging capacity •↓ H_2_O_2_, MDA, and EL	Habibi et al., 2019, 2020
Pomegranate fruit (*Punica granatum* L.)	4°C, 90 days	Chilling tolerance	•↑ ASC, phenols, and anthocyanins; ↑ DPPH scavenging capacity •↓ MDA, and EL	Nazoori et al., 2020
Persimmon fruit (*Diospyros kaki* Thunb.)	2°C, 45 days	Chilling tolerance, delays senescence	•↑ TSS, ASC, phenols, and flavonoids; ↑ activities of SOD, CAT, APX, PAL, PPO, and DPPH scavenging capacity •↓ H_2_O_2_, MDA, EL; ↓ activities of PG and PME	Niazi et al., 2021
Aonla fruit (*Emblica officinalis* Gaertn.)	5°C, 24 days	Chilling tolerance, delays senescence	↑ GABA, Pro, phenols, ASC, flavonoids, GSH Pro, ATP, and ADP; ↑ activities of GAD, GABA-T, P5CS, OAT, PAL, SOD, CAT, APX, and POD ↓ TSS, EL, MDA, H_2_O_2_, O_2_^•−^; ↓ PPO activity	Ali et al., 2022
Cut anthurium flowers (*Anthurium andraeanum* L.)	4°C, 3 weeks	Chilling tolerance	•↑ Pro, phenols, GB, and unSFA/SFA; ↑ activities of GABA-TP, SOD, CAT, APX, and GR; ↑ PAL/PPO activity ratio, and DPPH scavenging capacity •↓ H_2_O_2_, MDA and EL; ↓ activities of GAD, PLD, and LOX	Aghdam et al., 2015, 2016a,2016b
Blueberry fruit (*Vaccinium corymbosum* L.	4°C, 2 weeks	Delays senescence	• Increases ASC, GSH, phenols, and flavonoids; ↑ activities of SOD, CAT, APX, GR, PAL, C4H, and 4CL •↓ H_2_O_2_	Ge et al., 2018
Cornelian cherry fruit (*Cornus mas* L.)	4°C, 3 weeks	Delays senescence, preserves quality	•↑ AA, phenols, flavonoids, and anthocyanins; ↑ activities of SOD, CAT, APX, and GR; ↑ PAL/PPO activity ratio, and DPPH scavenging capacity •↓ Activities of LOX, PG, and PME; ↓ H_2_O_2_, MDA, and EL.	Aghdam et al., 2019; Rabiei et al., 2019
Mushrooom [*Agaricus bisporus* (J.E. Lange) Imbach]	4°C, 15 days	Retards cap browning, preserves nutritional and sensory quality	•↑ *GAD* activity; ↑ *PAL* expression and corresponding activity; ↑ ASC, phenols, and DPPH scavenging activity •↓ *GABA-T* expression, *PPO* expression and corresponding activity, MDA	Shekari et al., 2021
Pear fruit (*Pyrus ussuriensis* Maxim.)	0°C, 180 days, then 20°C, 12 days	Browning resistance	•↑ Expression of *AOX*, *SOD*, and *CAT* and corresponding activities •↓ ROS, and MDA	Li et al., 2019
Mango fruit (*Mangifera indica* L.)	15°C, 4 weeks	Preserves quality	•↑ ASC, phenols, and flavonoids; ↑ CAT activity, and DPPH scavenging capacity •↓ PPO activity	Rastegar et al., 2019
Apple fruit (*Malus* × *domestica* Borkh. “Cripps Pink”)	RT, 10 weeks	Preserves titratable acidity and quality	•↑ Expression of *GAD1*,2, *GABA-T1,2*, and *SSADH*, but *GAD3* unaffected •↑ Succ, and malate; ↑ activities of cytNAD-MDH, and PEPC; ↓ activities of cytNADP-ME, and PEPCK •↓ Respiration; ↓ ethylene, expression of *ACS*, *ACO*, and *ERF* before climacteric	Han et al., 2018
Apple fruit (“Honeycrisp”)	Conditioned at 10°C for 1 week, followed by 3°C for 5 months	Decreases soft scald, bitter pit or senescent breakdown		Al Shoffe et al., 2021
Pear fruit (*Pyrus pyrifolia* Nakai)	4°C, 4 weeks or 25°C, 3 days	Resistance to blue mold rot (*Penicillium. expansum*)	•↑ CAT activity; ↑ expression of *CHI, BGLU*, *PAL*, *POD*, and *PPO*, and corresponding activities	Yu et al., 2014; Fu et al., 2017
Orange fruit (*Citrus* × *sinensis* [L.] Osbeck)	RT, 80 days	Delays fruit rot	•↑ Glu, Pro, and citrate; ↑ expression of *GABA-T*, and *GABP* at 80 days •↓ Expression of *GAD2*, but not *GAD1*, at 20–80 days	Sheng et al., 2017
Tomato fruit (*Solanum lycopersicum* L.)	25°C, 36 h	Resistance to *Alternaria* rot (*Alternaria alternata*)	•↑ Expression of *GABA-TP1*, *SSADH*, *HXK*, and *PK*; ↑ activities of SDH, and MDH; ↑ ATP; ↑ expression of *SOD*, and *CAT*, and corresponding activities • Triggers SA signaling pathway and SAR; ↑ expression of *NPR1*, and *TAG1*; ↑ expression of *BGLU* and corresponding activity	Yang et al., 2017
Apple fruit (“Golden Delicious”)	RT, 8 days	Blue mold resistance (*Penicillium. expansum*)	•↑ GABA, and pyruvate; ↑ H_2_O_2_ (53 μmol g^–1^ FM), ASC, and GSH; ↑ activities of SOD, NADPH ox, CAT, GR, APX, DHAR, and MDHAR; ↑ activities of GAD, GDH, and GS; ↑ expression of *MT, MS, SAMS, SAMDC, ODC, ADC*, and *SPDS* •↓ activities of GABA-T, and SSADH; ↓ expression of *PAO*, and *DAO*	Zhu et al., 2022
Walnut kernel (*Juglans regia* L.)	20°C, 18 weeks	Attenuates browning and oxidative rancidity	•↑ unSFA/SFA ratio, phenols, oleic acid, linoleic acid, and linolenic acid; ↑ PAL/PPO activity ratio, and DPPH scavenging capacity •↓ H_2_O_2_, MDA, palmitic acid, stearic acid, and LOX activity	Ebrahimzadeh et al., 2019

*Symbols: ↑, increases; ↓, decreases.*

*ABALDH, 4-aminobutanal dehydrogenase; ACO, 1-aminocyclopropane-1-carboxylate oxidase; ACS, 1-aminocyclopropane-1-carboxylate synthase; ADC, arginine decarboxylase; ADP, adenosine diphosphate; AEC, Adenylate energy charge; AOX, alternative oxidase; APX, ascorbate peroxidase; ASC, ascorbate; ATP, adenosine triphosphate; BGLU, β-1,3-glucanase; C4H, cinnamate-4-hydroxylase; CAT, catalase; CHI, chitinase; 4CL, 4-coumarate/coenzyme A ligase; cyt, cytosolic; DAO, diamine oxidase; DHAR, dehydroascorbate reductase; DPPH, 2,2-diphenyl-1-picryl-hidrazil; EL, electrolyte leakage; ERF, ethylene-responsive factor; FRAP, ferric reducing antioxidant potential; GABA, γ-aminobutyric acid; GABA-TP or GABA-TOG, pyruvate-or 2-oxoglutarate-dependent GABA transaminase; GAD, glutamate decarboxylase; GABP, GABA permease; GB, glycine betaine; GDH, glutamate dehydrogenase; Glu, glutamate; GR, glutathione reductase; GS, glutamine synthetase; GSH, reduced glutathione; GPX, glutathione peroxidase; GST, glutathione S-transferase; H_2_O_2_, hydrogen peroxide; HXK, hexokinase; LOX, lipoxygenase; MDA, malondialdehyde; MDH, malate dehydrogenase; MDHAR, monodehydroascorbate reductase; ME, malic enzyme; MS, methionine synthase; MT, metallothionein; NADH, reduced dinucleotide; NADPH, reduced dinucleotide phosphate; NADPH Ox, NADPH oxidase; NPR, non-inducible pathogenesis-related; O_2_, superoxide anion; OAT, ornithine δ-aminotransferase; ODC, ornithine decarboxylase; P5CS, Δ^1^-pyrroline-5-carboxylate synthetase; PAL, phenylalanine ammonia lyase; PAO, polyamine oxidase; PDH, proline dehydrogenase; PEPC, phosphoenolpyruvate carboxylase; PEPCK, phosphoenolpyruvate carboxykinase; PG, polygalacturonase; PK, pyruvate kinase; PLD, phospholipase D; PME, pectin methylesterase; POD, peroxidase; PPO, polyphenol oxidase; PR, pathogenesis-related; Pro, proline; ProDH, proline dehydrogenase; Put, putrescine; RT, room temperature; SA, salicylate; SAM, S-adenosylmethionine; SAMS, S-adenosylmethionine synthetase; SAMDC, S-adenosylmethionine decarboxylase; SAR, systemic acquired resistance; SDH, succinate dehydrogenase; SFA, saturated fatty acids; SOD, superoxide dismutase; Spd, spermidine; SPDS, spermidine synthase; Spm, spermine; SSADH, succinic semialdehyde dehydrogenase; TAG, TAG transcription factor; TSS, total soluble sugars.*

Together, these studies suggest that elevated endogenous GABA or exogenous GABA maintains the quality of apple fruit by stimulating the activity of the GABA shunt and the synthesis of malate, and delaying fruit ripening. Notably, the inhibition of ethylene-mediated ripening by 1-methylcyclopropene increases the GABA level in “Empire” and “Honeycrisp” apples and in “AC Harrow Crisp” pears stored under LT, CA conditions (Deyman et al., 2014a; Lum et al., 2016a; Flaherty et al., 2018). While the interaction between ethylene and GABA biosynthesis requires further study, exogenous GABA seems to elicit similar responses as LT, CA conditions.

### Exogenous GABA Alleviates Chilling Injury, Bacterial/Fungal Decay, and Loss of Quality

The attenuation of LT injury in peach, banana, orange, pomegranate, persimmon and aonla fruits, as well as cut anthurium flowers, by exogenous GABA is evident from the preservation of membrane fluidity and stability (decrease in electrolyte leakage), which is accompanied by decreases in reactive oxygen species (ROS; e.g., hydrogen peroxide and superoxide radical), greater antioxidant and radical-scavenging capacities, the maintenance of intracellular ATP and NADH, and the accumulation of potential osmolytes (i.e., soluble sugars, PAs and proline) (Shang et al., 2011; Yang et al., 2011; Wang Y. et al., 2014; Aghdam et al., 2015, 2016a,2016b; Habibi et al., 2019, 2020; Nazoori et al., 2020; Niazi et al., 2021; Ali et al., 2022; Table 3).

Similar mechanisms are involved in: the delay of senescence and preservation of quality in LT-stored blueberries, cherries and mushrooms, RT-stored apples, and conditioned LT-stored apples (Ge et al., 2018; Han et al., 2018; Aghdam et al., 2019; Rabiei et al., 2019; Al Shoffe et al., 2021; Shekari et al., 2021); browning resistance and the preservation of quality in LT-stored pear and mango (Li et al., 2019; Rastegar et al., 2019); resistance against fungal infection in LT-or RT-stored pear, orange, strawberry and tomato fruits (Yu et al., 2014; Fu et al., 2017; Sheng et al., 2017; Yang et al., 2017); and resistance against various pathogens and browning in RT-stored walnut kernels (Ebrahimzadeh et al., 2019; Table 3). Notably, pathogen resistance is promoted by salicylate signaling and disease resistance proteins, and maintaining the integrity of the cell wall barrier (Yu et al., 2014; Fu et al., 2017; Yang et al., 2017; Gao et al., 2018a; Zhao et al., 2021; Table 3), and the loss of apple fruit acidity is retarded by accumulating malate and suppressing ethylene biosynthesis (Han et al., 2018; Table 3).

Hou et al. (2022) have shown that the fresh-cut process does not affect the organoleptic quality of carrots stored under LT for hours, though it appears to enhance GABA biosynthesis from both Glu and PAs (Table 4). This result is consistent with the previously reported impact of wounding/mechanical damage on GABA accumulation (Shelp et al., 2012a). Notably, the resistance to browning and bacterial pathogens in fresh-cut pear, apple and potato during prolonged LT storage is improved by both CA and exogenous GABA *via* the mechanisms described above (Gao et al., 2018a,b; Wang D. et al., 2021; Zhao et al., 2021).

**TABLE 4:** The postharvest marketability of fresh-cut horticultural commodities is improved by low temperature, controlled atmosphere conditions or exogenous GABA.

Commodity	Storage conditions	Treatment	Marketability	Biochemical and molecular responses	References
Carrot root (*Daucus carota* L.)	4°C for 9 h		Organoleptic quality unaffected	•↑ GABA; ↑ expression of *GAD1, GAD2, GABA-T2* and *PAO*; ↑ activities of GAD, DAO, PAO, and ABALDH •↓ Glu, Put, Spd, and Spm; ↓ *GABA-T1* expression; ↓ GABA-T activity	Hou et al., 2022
Pear fruit (*Pyrus pyrifolia* (f. Burm.) Nakai)	5°C, 10 kPa CO_2_ + 11 kPa O_2_, 6 days		Alleviates browning and preserves quality	•↑ GABA and Pro; ↑ activities of GAD, GABA-T, P5CS, and OAT; ↑ linoleic acid (unSFA/SFA) •↓ activities of PDH, PLD, and LOX; ↓ palmitic, oleic acid, and stearic acid; ↓ EL and MDA	Wang D. et al., 2021
Apple fruit (*Malus* × *domestica* Borkh. “Fuji”)	4°C, 6 days	GABA	Resistance to various bacterial pathogens and browning	•↑ expression of *CAT*, *PAL*, *CHI*, and *BGLU* and corresponding activities •↑ expression of genes associated with caffeic acid, lignin, anthocyanin and coumarate biosynthesis; ↑ expression of *XTHs*, *PEI*s, *Ces*, *EXTs*, and *PRP*s •↓ O_2_^•⁣–^ and H_2_O_2_ •↓ phenols, flavonoids, and soluble pectin; ↓ expression of a *lacasse* gene; PPO expression unaffected	Gao et al., 2018a; Zhao et al., 2021
Potato tuber (*Solanum tuberosum* L.)	4°C, 6 days	GABA	Browning resistance	•↑ SOD and CAT activities •↓ PPO activity, O_2_^•⁣–^, H_2_O_2,_ and MDA	Gao et al., 2018b

*Symbols: ↑, increases; ↓, decreases.*

*XTH, xyloglucan endotransglucosylase/hydrolase; PEI, pectin esterase inhibitor; Ces, cellulose synthase; Ext, extensin; PRP, proline-rich protein; remaining abbreviations are given in Table 3.*

In summary, the application of exogenous GABA to postharvest fruits, vegetables (including mushrooms), cut flowers, and walnuts delays senescence, attenuates chilling injury and fungal/bacterial-induced decay, and helps to preserve sensory and nutritional quality. GABA can promote activities of the GABA shunt, and the TCAC, antioxidant, secondary and phytohormone pathways, which in turn, reduce the stress-induced ROS level. However, the precise mechanisms whereby GABA interacts with other signaling molecules such as Ca^2+^, H_2_O_2_, PAs, salicylic acid, nitric oxide and melatonin, or with phytohormones such as ethylene, abscisic acid and auxin remain unknown (Bor and Turkan, 2019; Podlešákova et al., 2019; Seifikalhor et al., 2019; Suhel et al., 2022).

## Prospects for Improving the Postharvest Marketability of Horticultural Commodities With Exogenous GABA

### Stomatal Functioning and Tolerance/Resistance to Drought and Pathogens

In cut flowers, excessive transpiration can result in a loss of turgor, premature wilting of flowers and leaves, and accelerate flower senescence. Water loss *via* the stomata can also result in a loss of FM and quality in leafy vegetables and immature green fruits. Therefore, it may be beneficial to manipulate endogenous GABA by applying exogenous GABA to restrict stomatal opening and prevent water loss (Xu et al., 2021a). Stomatal closure may also aid in preventing bacterial and fungal pathogens from entering leaves or fruits (Gahir et al., 2021). Thus, regulation of stomatal function may be a promising strategy for improving postharvest quality and safety of horticultural products (van Meeteren and Aliniaeifard, 2016).

### Vacuolar Functioning and Flavor

Malate is the predominant organic acid in ripe apple fruit, and most of this is found in the vacuole. The transport of malate across the apple tonoplast is probably mediated by the apple ALMT9 (*Md*Ma1) (Li et al., 2020; Table 2). Both the full-length protein, *Md*Ma1, and its naturally occurring truncated protein, *md*ma1, localize to the tonoplast; when expressed in *Xenopus laevis* oocytes and *Nicotiana benthamiana* cells, *Md*Ma1 mediates a malate-dependent inward-rectifying current, whereas the ma1-mediated transmembrane current is much weaker, indicating that ma1 has significantly lower malate transport activity than Ma1. RNA interference suppression of *MdMa1* expression in “McIntosh” apple leaves, “Empire” apple fruit, and “Orin” apple calli significantly decreases the malate level. Notably, the most highly-related ortholog in *Arabidopsis*, ALMT9, transports mainly Cl^–^ into the vacuole, but is subject to negative regulation by cytosolic GABA (Bown and Shelp, 2016; Xu et al., 2021a,b). Thus, the application of GABA on apple fruit during LT storage could reduce malate accumulation and the acidity of apple fruits. Bai et al. (2015) have suggested that a major network of genes, including *MdALMT9*, is associated with the developmental regulation of apple fruit acidity in “Golden Delicious,” but such a network has not been investigated during the ripening period (Ban and Xu, 2020). It could have implication for breeding apples or other fruits in order to preserve or enhance their flavor during postharvest storage.

Tomato SlCAT9 encodes a tonoplast Glu/Asp/GABA exchanger and its expression increases in tomato fruit during ripening (Snowden et al., 2015; Table 2). Such an exchanger might provide a mechanism for remobilizing GABA from the vacuole during cellular Glu uptake (Chung et al., 1992). On the other hand, ripening-specific overexpression of *SlCAT9* increases the accumulation of GABA, Glu and Asp by approximately 20-, one- and sixfold, respectively (Snowden et al., 2015). Notably, greater Glu and Asp accumulation in the vacuole contribute to umami taste development in tomato fruit during ripening (Takayama and Ezura, 2015). Elevated GABA accumulation in the vacuole of immature fruit might deter insect pests and pathogens, whereas lower GABA accumulation in tomato fruit during ripening might be beneficial for attracting insects and animals for successful seed dispersal (Takayama and Ezura, 2015; Shelp et al., 2021). The properties of apple CAT9 have not yet been characterized (Table 2), but they could have implications for altering the development, flavor and biotic resistance of apple fruits.

## Safety and Commercial Production of GABA

Natural GABA is ubiquitous in plants and animals, and exogenous GABA is readily catabolized (Tuin and Shelp, 1994; Hijaz and Killiny, 2019; Oketch-Rabah et al., 2021). Nevertheless, the application of exogenous GABA to horticultural commodities during postharvest storage is likely to result in GABA accumulation. GABA is marketed worldwide as a dietary ingredient, food supplement and medicinal agent/drug. Available evidence suggests that GABA ingestion is not associated with adverse health events, probably due to the inability of GABA to cross the human blood–brain barrier (Boonstra et al., 2015; Oketch-Rabah et al., 2021). Also, GABA meets the statutory requirement of reasonable certainty of no harm to the environment (The United States Environmental Protection Agency, 2004).

Large scale commercial production of GABA would be necessary to support its use in postharvest storage of horticultural commodities. While chemical synthesis of GABA is feasible, this process requires expensive and hazardous reagents and generates unwanted by-products (Grewal, 2020; Oketch-Rabah et al., 2021). GABA can be formed from Glu using purified GAD and the coenzyme pyridoxal-5’-phosphate, but the purification of GAD is expensive and the enzyme tends to be unstable. The preferred manufacturing method for commercial production of GABA is fermentation by lactic acid bacteria because of their GRAS (Generally Recognized As Safe) status, high stress tolerance, and ability to release GABA into the extracellular matrix (Grewal, 2020; Jin et al., 2021; Laroute et al., 2021; Yogeswara et al., 2021).

## Concluding Remarks

Research on the postharvest physiological, biochemical, and molecular responses of horticultural commodities to LT and CA storage provides valuable information for conceiving new strategies to improve their marketability. These storage conditions are generally associated with the promotion of GABA pathway activity, with or without the accumulation of GABA, delaying senescence, preserving quality and ameliorating chilling injury. Induction and co-ordinated gene expression, together with the biochemical properties and subcellular location of the corresponding encoded proteins, suggest that *MdGAD1,2*, *MdGABA-T1,2, MdSSADH1, MdCuAO1*, and *MdALDH10A8*,9 are important determinants of GABA pathway activity in stored apple fruits, regardless of the storage condition. Notwithstanding, the targeted metabolite profiles suggest that protein hydrolysis, Ca^2+^/CaM activation or H^+^ stimulation of GAD activity, and changing redox balance are especially significant under LT, CA conditions. Furthermore, flux estimates suggest that the GABA pool is primarily derived from Glu, rather than PAs, and that SSA is converted mainly to succinate, rather than GHB.

Exogenous GABA is a promising strategy for promoting the level of endogenous GABA and the activity of the GABA shunt, which results in increased carbon flux through respiratory pathways, leading to elevated levels of NADH, NADPH and ATP (Aghdam et al., 2018, 2020; Shelp et al., 2021). Adequate ATP and NADPH are essential for: (i) fortifying the activity of ROS avoidance and scavenging systems; (ii) promoting the accumulation of endogenous proline and PAs; (iii) promoting the activity of secondary pathways, which results in the generation of salicylate for promoting the expression and activity of PR proteins, as well as phenols, flavonoids, and anthocyanins for scavenging radicals; (iv) limiting the activity of phospholipase D and lipoxygenase, resulting in increased membrane stability and fluidity; and (v) enhancing NADPH oxidase activity for triggering H_2_O_2_ accumulation. As a result, chilling injury and fungal/bacterial decay are deterred during postharvest storage, delaying senescence, preserving nutritional quality, and improving the postharvest marketability of horticultural crops. The occurrence of the tonoplastic ALMT presents the opportunity to restrict transpirational water loss by applying exogenous GABA to negatively regulate malate influx into the vacuole and light-induced stomatal opening in cut flowers and immature green fruit. Also, both the ALMT transporter and tonoplast CAT exchanger present the opportunity to manipulate fruit flavor. Available evidence suggests that exogenous GABA does not adversely affect human or environment health, though further optimization of microbial fermentation is probably necessary to ensure an adequate commercial supply of GABA for use as a biostimulant in the postharvest storage of horticultural commodities.

## Author Contributions

MA conceived and wrote the original manuscript, prepared original figures, and reviewed the revised manuscript. EF conducted the bioinformatics analysis, and reviewed the original and revised manuscripts. BS conceived and administered the project, and revised the original manuscript and figures. All authors have read and agreed to the published version of the manuscript.

## Conflict of Interest

The authors declare that the research was conducted in the absence of any commercial or financial relationships that could be construed as a potential conflict of interest.

## Publisher’s Note

All claims expressed in this article are solely those of the authors and do not necessarily represent those of their affiliated organizations, or those of the publisher, the editors and the reviewers. Any product that may be evaluated in this article, or claim that may be made by its manufacturer, is not guaranteed or endorsed by the publisher.
